# Application and interpretation of multiple statistical tests to evaluate validity of dietary intake assessment methods

**DOI:** 10.1186/s12937-015-0027-y

**Published:** 2015-04-22

**Authors:** Martani J Lombard, Nelia P Steyn, Karen E Charlton, Marjanne Senekal

**Affiliations:** 1 Centre of Excellence for Nutrition (CEN), North-West University, Private Bag X6001, Nutrition, Box 594, Potchefstroom, 2520 South Africa; 2 Division of Human Nutrition, University of Cape Town, Cape Town, South Africa; 3 School of Medicine, Faculty of Science, Medicine and Health, University of Wollongong, Wollongong, Australia

**Keywords:** Validity, Dietary intake assessment, Food frequency questionnaire, 24-hour recall, Statistical tests, Agreement, Association, Bias

## Abstract

**Background:**

Several statistical tests are currently applied to evaluate validity of dietary intake assessment methods. However, they provide information on different facets of validity. There is also no consensus on types and combinations of tests that should be applied to reflect acceptable validity for intakes. We aimed to 1) conduct a review to identify the tests and interpretation criteria used where dietary assessment methods was validated against a reference method and 2) illustrate the value of and challenges that arise in interpretation of outcomes of multiple statistical tests in assessment of validity using a test data set.

**Methods:**

An in-depth literature review was undertaken to identify the range of statistical tests used in the validation of quantitative food frequency questionnaires (QFFQs). Four databases were accessed to search for statistical methods and interpretation criteria used in papers focusing on relative validity. The identified tests and interpretation criteria were applied to a data set obtained using a QFFQ and four repeated 24-hour recalls from 47 adults (18–65 years) residing in rural Eastern Cape, South Africa.

**Results:**

102 studies were screened and 60 were included. Six statistical tests were identified; five with one set of interpretation criteria and one with two sets of criteria, resulting in seven possible validity interpretation outcomes. Twenty-one different combinations of these tests were identified, with the majority including three or less tests. Coefficient of correlation was the most commonly used (as a single test or in combination with one or more tests). Results of our application and interpretation of multiple statistical tests to assess validity of energy, macronutrients and selected micronutrients estimates illustrate that for most of the nutrients considered, some outcomes support validity, while others do not.

**Conclusions:**

One to three statistical tests may not be sufficient to provide comprehensive insights into various facets of validity. Results of our application and interpretation of multiple statistical tests support the value of such an approach in gaining comprehensive insights in different facets of validity. These insights should be considered in the formulation of conclusions regarding validity to answer a particular dietary intake related research question.

## Background

Validation of a dietary intake assessment method is the process of determining the accuracy by which the method measures actual dietary intake over a specified time period [1,2]. Most often, dietary assessments attempt to measure usual or habitual intake [3]. Validation of the dietary intake assessment method used is required in order to demonstrate the magnitude and direction of measurement error, potential causes of the measurement error, and to identify ways in which these errors may be minimized or accounted for in the analyses [4]. Validation also provides information on possible misclassification, which is especially relevant when diet-disease associations are investigated in epidemiological studies [1].

The process of validating dietary intake methodology is complex and relies on the ability of participants to provide accurate dietary intake information [5]. Various other factors that also influence the validation process include the type of dietary assessment method used, the type of reference method used (i.e. biomarker, doubly labelled water or another dietary assessment method), the sample size and characteristics of the population included in the validation study, seasonality, and the sequence of data collection (whether the reference method is applied in a random order or not) [6]. The ideal procedure would be to determine absolute validity, thus whether the measure accurately reflects the exact concept that it is intended to reflect [3]. For this purpose a perfect or near perfect indicator of the target concept, referred to as a gold standard (criterion), is needed [3]. However, because of the lack of a gold standard in dietary assessment methodology, the degree of measurement error in the estimation of usual dietary intake cannot be accurately determined [1,7,8].

Unless direct observation techniques are employed, validation studies cannot compare a test method with the absolute truth. However, the test method could be compared with a reference method that measures the same underlying concept over the same time period (relative validity). Ideally the reference method should have been shown to have a degree of demonstrated validity, although not necessarily providing an exact measure of the truth [3,8]. Both the test and reference methods inherently have some degree of inaccuracy and internal measurement errors and the two methods must thus be independent in order to avoid a correlation of error. For instance, a quantitative food frequency questionnaire (QFFQ) method that relies on memory can be validated against weighed records that do not require the subject to recall their intake [3,8,9]. However, poor validity of the test method may not necessarily be attributable to errors associated with the method, but may also be related to errors associated with the reference method [6,10]. To account for this possibility, a third criterion method such as a biomarker or doubly labelled water is often used to triangulate error, [10] but inclusion of such a criterion method is often expensive and not logistically possible [8].

Once dietary intake data has been generated using both methods, various statistical tests such as correlation coefficients and Bland-Altman analyses can be applied in the assessment and interpretation of validity of the test method. These statistical tests reflect different facets of validity such as agreement, association, or bias at group or individual level [8]. There is, however, no consensus on the type and number of statistical tests that should ideally be applied to assess validity of a dietary intake assessment method [6]. From a theoretical point of view it could be argued that conducting multiple tests that reflect different facets of validity would provide superior insights into the validity of the test method. Interpretation of multiple statistical tests may, however, prove to be challenging. It is for example plausible that the outcome of one test may support validity of the test method for a particular facet of validity, for instance agreement at either individual or group level, while the same test may reflect poor validity for another facet, for instance association or bias on either individual or group level.

The aims of this paper were firstly to conduct a review of the literature to identify the range of statistical tests and interpretation criteria used in studies where a QFFQ was validated against a reference method (relative validity). Secondly, we wanted to investigate the value of and challenges that may arise in the interpretation of the outcomes of multiple statistical tests in the assessment of the relative validity of a QFFQ (test method) using data on total energy intake and intake of selected nutrients derived from a test data set.

## Methods

### In-depth literature review

An in-depth literature review was conducted and four databases (EBSCOhost, Pubmed, Google Scholar and Science Direct) were accessed to identify papers that reported on the validation of a dietary assessment method against another dietary assessment method. Poster and conference proceedings were not included in the search. Search terms included “validation, validity, reliability, repeatability, dietary assessment, food frequency questionnaire, 24-hour recall, weighed food record, agreement, association and bias”. Relevant studies published between January 2009 and December 2014 were identified. Review papers, studies that used biomarkers as part of the validation process, and duplicate articles were excluded. Individual statistical tests and combinations of tests used in the studies included in the review were recorded and ranked according to frequency of use of specific combinations. The interpretation criteria for each identified statistical test and facets of validity reflected by the test were critically reviewed and recorded.

### Test data set

The test data set consisted of dietary data that was collected from a convenience sample of 47 adults (18–65 years old; 76.6% female) residing in a rural area in the Eastern Cape (South Africa) by means of a newly developed QFFQ (test method). The QFFQ comprised 21 maize-based cultural specific food items and beverages and was accompanied by a portion size food photograph series [11,12]. The recall period of the QFFQ was the past month and response categories included “less than once a month”, “amount per month”, “amount per week” and “amount per day”. Four non-consecutive 24-hour recalls (including one weekend day) were also conducted with each participant over a one month period (reference method). The test method was administered before the reference method. For illustration of the value of and challenges that may arise in the interpretation of the outcomes of multiple statistical tests total energy, fat, protein, carbohydrate, iron, folate and vitamin A intakes were derived from the QFFQ and 24-hour recalls using the South African dietary analyses software, FoodFinder 3 [13]. These variables were compared using the identified statistical tests and validity interrogated using the interpretation criteria, for the test and reference method results. Ethical approval was obtained from the Research Ethics Committees of the University of Cape Town (UCT) (FHS-HREC 123/2003).

## Results

### Number of studies included in the review and summary of identified statistical tests and test combinations used

A total of 102 papers were screened of which 60 were included in the review, while 42 were excluded for the reasons mentioned in the methods. Six different statistical tests were identified, five with one set of interpretation criteria each and one with two sets of criteria (cross-classification in the same or opposite tertiles), resulting in a total of seven possible validity interpretation outcomes (Table 1). The most commonly used test was the correlation coefficient (57 studies, 18 combinations), followed by cross-classification (28 studies, 12 combinations), Bland Altman analyses (27 studies, 10 combinations), *t*-test or Wilcoxon signed rank test (22 studies, 7 combinations), weighted Kappa coefficient (15 studies, 9 combinations) and percent difference (5 studies, 4 combinations) (86.). Twenty-one different combinations of the six statistical tests were identified in the 60 studies. The majority of combinations included three or fewer tests, with the coefficient of correlation featuring as a single test (delineated as a “combination” in Table 2) and in all but three of the remaining 20 combinations. Bland Altman analyses and cross-classification were included in approximately half of the combinations, with the weighted Kappa coefficient used less often. The least used test in combinations seems to be the percent difference (Table 2). Not one of the reviewed studies that included Bland Altman analyses considered the clinical importance of the width of limits of agreement (LOA) in their discussion and conclusions regarding the validity of the method being tested. Furthermore, all studies concluded that the test dietary assessment method was valid for use in the respective populations.

**Table 1:** **Summary of identified statistical tests and interpretation criteria for validation of dietary intake assessment methods**

Statistical test	Facet of validity reflected	Interpretation criteria		
		Good outcome	Acceptable outcome	Poor outcome
Correlation coefficient [8,14-16,18,28-67,90]	Strength and direction of association at individual level [8]	≥0.50 [2]	0.20 - 0.49 [2]	<0.20 [2]
Paired *t*-test/ Wilcoxon signed rank test [8,22,23,25,27,28,33,34,36,48,49,52-56,60,62,65,66,68,69,91]	Agreement at group level [8]	P > 0.05 [8]		P ≤ 0.05 [8]
Percent difference [8,22,23,25,27,28,33,34,49,52-56,60,65-72,91]	Agreement at group level (size and direction of error) [8]		0.0 - 10.0% [77]	>10%
Cross-classification (tertiles/ quartiles or quintiles) [8,22,31,32,35-38,41,42,44-51,55-61,63-69,91]	Agreement (including chance), at individual level [8]	≥50% in same tertile/quartile [2] ≤10% in opposite tertile/quartile [2]		<50% in same tertile/quartile [2] >10% in opposite tertile/quartile [2]
	• In same tertile			
	• In opposite tertile			
Weighted Kappa statistics (coefficient) [8,24,26,30,40,43,54,58,59,63,64,66-69,91]	Agreement (excluding chance) at individual level [8]	≥0.61 [2]	0.20 - 0.60 [2]	<0.20 [2]
Bland Altman analysis: Correlation between mean and mean difference) [6,21,33,34,37-39,43,50,53,54,61,63,69,76,92]	Presence, direction and extent of bias at group level [6,76]	P > 0.05 [6]		P ≤ 0.05 [6]

**Table 2:** **Summary of statistical test combinations applied in reviewed validation studies**

Combination of tests ranked from most to least frequent use	Statistical test (number of combinations						Number of studies the combination (total n = 60)	References for identified studies
	Correlation coefficient	*t* -test/ Wilcoxon	Cross-classification	% difference	Kappa Statistic	Bland Altman		
1	X	X					8	[17,22,23,25,36,51,52,55]
2	X						7	[14,16,18,29,35,37,38]
3	X		X			X	6	[32,39,42,44,47,61]
4	X	X				X	5	[21,27,28,33,62]
5	X					X	5	[19,20,34,48,53]
6	X		X				5	[15,31,38,41,45]
7	X		X		X		4	[58,59,63,64]
8	X	X	X				2	[49,65]
9	X	X	X		X	X	2	[50,54]
10	X		X	X		X	2	[46,68]
11	X				X		2	[24,26]
12	X	X			X	X	2	[43,69]
13	X	X	X			X	2	[56,60]
14	X		X	X			1	[57]
15	X				X	X	1	[30]
16	X			X	X		1	[40]
17	X		X		X	X	1	[67]
18	X	X	X		X		1	[66]
19			X		X	X	1	[92]
20				X			1	[90]
21			X				1	[91]

### Explanation of identified tests, facets of validity reflected and suggested interpretation criteria

Details regarding the identified tests, interpretation criteria and facets of validity reflected are as follows (detail of interpretation criteria are presented in Table 1 and are not repeated in the text):

Correlation coefficients (Pearson, Spearman or Interclass) are widely used in validation studies and measure the strength and direction of the association between the two different measurements at individual level [8,14-69]. They do, however, not measure the level of agreement between the two methods. In cases where more than one questionnaire is used, for instance multiple weighed records or 24-hour recalls, de-attenuated correlation coefficients can be used to adjust for day-to-day variation [32]. Correlation coefficient values can range between −1 (perfect negative correlation) and 1 (perfect positive correlation), with a coefficient of zero reflecting no linear relationship between the two measurements [70]. Because correlation coefficients do not provide any insight into the level of agreement between two measurements, [8,71,72] it is not appropriate to use these tests as the sole determinant of validity [73].

The paired *T*-test or Wilcoxon signed rank test reflects agreement between two measures at group level [74,75]. Assessment of mean percent difference between the reference and test measure reflects agreement at group level (size and direction of error at group level) [76,77]. For calculation of the mean percentage difference the reference value is subtracted from the test measure value, divided by the reference measure and multiplied by 100 for each participant [74,75]. The mean percentage difference is then calculated for the total sample.

Cross-classification of participants for both the test and reference methods into categories, usually according to tertiles, quartiles or quintiles depending on the sample size, allows calculation of the percentage of participants correctly classified in the same category and the percentage misclassified in the opposite category [2,8,78]. Accurate classification is important and indicates to what extent the dietary intake assessment method is able to rank participants correctly, this reflects agreement at individual level [79]. Ranking of dietary intake data is especially important in the investigation of diet-disease associations [8,80]. However, cross-classification of data is limited in that the percentage of agreement includes chance agreement [8].

The weighted Kappa coefficient is typically used for data that are ranked into categories or groups and excludes chance agreement [2,8,10]. The magnitude of weighted Kappa coefficient values are mostly determined by factors such as the weighting applied, as well as the number of categories included in the scale [80]. Weighted Kappa coefficient values range from −1 to 1 with values between 0 and 1 generally being expected [81]. Values of zero or close to zero can be considered as an indication of “*no more than pure chance*”, while negative values indicate agreement “*worse*” than can be expected by chance alone [80]. The weighting of the Kappa coefficient depends on the number of categories or groups, for instance if there are three categories, a score of 1 is allocated to participants in the same group, 0.5 for those in adjacent groups and 0 for those in opposite groups [80]. The Kappa coefficient does not take into account the degree of disagreement between methods and all disagreement is treated equally as total disagreement. It also does not indicate whether agreement or lack thereof is because of a systematic difference between the two methods, or because of random differences (error because of chance) [80].

Bland-Altman analysis reflects the presence, direction and extent of bias, as well as the level of agreement between two measures at group level [10]. Spearman correlation coefficients are calculated between the mean of the two methods and the mean difference of the two methods to establish the association between the size of the error (or difference between the two methods) and the mean of the two methods, which reflect the presence of proportional bias as well as the direction thereof [8,10,72,82]. If proportional bias is present i.e. as the mean intake becomes larger, so does the difference in one direction, the Spearman rank correlation coefficient between the mean intakes and the difference between intakes will be significant [72].

Bland-Altman analysis includes plotting the difference between the measurements (test - reference measure) (y-axis) against the mean of the two measures [(test measure + reference measure / 2)] (x-axis) for each subject to illustrate the magnitude of disagreement, identify outliers and trends in bias [8,72,76,83]. The LOA [95% confidence limits of the normal distribution] are calculated as the mean difference ± 1.96 SD [72,84] and reflect over and underestimation of estimates [72]. It is important to note that Bland and Altman [83] indicated that “the decision about what is acceptable agreement is a clinical one; statistics alone cannot answer the question.”

### Illustration of the application of identified statistical tests and interpretation criteria using a test data set

The mean (SD) and median (IQ range) estimates for energy and nutrient intakes derived from the test data set are presented in Table 3 (not alluded to in the discussion section). Key outcomes of the application of the six statistical tests and seven interpretation criteria (two for cross classification) for the assessment of the relative validity of these variables as follows (Table 4):

**Table 3:** **Mean(SD) and median(IQ Range) estimates for energy and select nutrient intakes derived from the test data set** ^**1**^

Nutrient	EER/RDA		Test method (g)		Reference method (g)		Difference (g)		
	Males	Females	Mean (SD)	Median (IQ Range)	Mean (SD)	Median (IQ Range)	Mean (SD)	95% CI of difference	Median (IQ Range)
Energy (kJ)	12881	10093	12463 (5854)	12475 (7686–17091)	13819 (3677)	13661 (11003–16225)	−1356 (6025)	−9192 - 8602	−1642 (−6439 - 2830)
Protein (g)	56	46	67.3 (34.0)	66.0 (44.7-91.3)	84.9 (26.2)	81.6 (68.8-99.5)	−17.6 (38.5)	−75.5-48.0	−19.3 (−42.4 - 4.2)
Fat (g)	118	93	69.5 (46.9)	54.8 (38.3 – 86.5)	83.9 (31.5)	82.8 (62.7-102.5)	−14.4 (55.5)	−83.3-62.1	−12.8 (−54.0-16.9)
Carbohydrates (g)	130	130	475.7 (222.3)	475.7 (289.1-618.3)	494.1 (148.6)	487.7 (397.3-572.0)	−18.5 (215.4)	−326.2-325.5	−16.3 (−153.1-140.3)
Folate (mcg)	400	400	558.0 (355.2)	454.0 (331.0-788.0)	419.6 (235.7)	405.8 (247.5-552.7)	138.4 (393.7)	−401.6-842.9	108.6 (0.9-305.0)
Vitamin A (mcg)	900	700	192.6 (231.1)	92.0 (51.0-242.0)	346.7 (276.9)	277.0 (171.1-402.4)	−154.2 (360.9)	−895.2-373.9	−105.2 (−278.8-5.1)
Iron (mg)	8	18	12.1 (7.1)	12.3 (6.5-15.7)	16.1 (5.5)	15.4 (11.4-0.2)	−6.0 (8.2)	−18.9 – 10.5	−6.2 (−11.1- -1.7)

^1^Energy and nutrient intake derived from a QFFQ and four repeated 24-hour recalls conducted in 18–65 year old adults (n = 47, 11 males & 36 females) in a rural area in the Eastern Cape.

RDA: Recommended dietary allowances.

SD = standard deviation, IQ Range = inter quartile range, CI = 95% Confidence interval.

**Table 4:** **Statistical test outcomes and interpretation for energy and nutrient intakes derived from the test data set** ^**1**^

Nutrient	Spearman correlation (r value)	Wilcoxon signed rank test (P value)	Percentage difference (%)	Cross-classification (Tertiles)		Weighted Kappa statistics (value)	Bland – Altman ^ 2,3 ^ Spearman Correlation (r value)
	Association (strength & direction)	Agreement	Agreement (size & direction of error)	Agreement (including chance)		Agreement (excluding chance)	Presence, direction and extent of bias
				% in same tertile	% in opposite tertile		
Level of validation	Individual	Group	Group	Individual	Individual	Individual	Group
Energy (kJ)	0.26	P > 0.05	−9.8	46.8	19.2	0.20	P < 0.001
Validly interpretation	Acceptable	Good	Good	Poor	Poor	Acceptable	Biased
Protein (g)	0.23	P < 0.01	−19.1	42.6	23.4	0.12	P < 0.05
Validly interpretation	Acceptable	Poor	Acceptable	Poor	Poor	Poor	Biased
Fat (g)	0.01	P > 0.05	−6.9	34.0	31.9	−0.01	P > 0.05
Validly interpretation	Poor	Good	Good	Poor	Poor	Poor	Not biased
Carbohydrates (g)	0.40	P > 0.05	−1.4	50.0	17.4	0.25	P < 0.01
Validly interpretation	Acceptable	Good	Good	Good	Poor	Acceptable	Biased
Folate (mcg)	0.40	P < 0.05	33.0	53.2	8.5	0.30	P < 0.01
Validly interpretation	Acceptable	Poor	Poor	Good	Good	Acceptable	Biased
Vitamin A (mcg)	0.15	P < 0.01	−22.9	34.0	14.9	0.03	P > 0.05
Validly interpretation	Poor	Poor	Poor	Poor	Poor	Poor	Not biased
Iron (mg)	0.38	P > 0.05	−24.8	51.1	23.4	0.29	P < 0.01
Validly interpretation	Acceptable	Good	Poor	Good	Poor	Acceptable	Biased

^1^Energy and nutrient intake derived from a QFFQ and four repeated 24-hour recalls conducted in 18–65 year old adults (n = 47, 11 males & 36 females) in a rural area in the Eastern Cape.

^2^% in LOA: Energy: 93.6%; protein: 95.7%, fat: 97.9%, carbohydrate: 95.7%; folate: 95.7%, vitamin A: 89.4%; iron: 98%.

^3^Upper & lower limits of agreement (LOA) and DRI for females aged 18 to 55: energy (kJ): −13406 & 10694 (EER: 10093 kJ); protein (g): −94.6 & 59.4 (RDA: 46 g); fat: −125.4 & 96.6 (no DRI); folate (mcg): −649 & 925.8 (RDA: 400mcg); vitamin A (mcg): −876 &576.4 (RDA: 700mcg); iron (mg): −22,4 &10.4 (RDA: ????mg).

*Interpretation criteria for statistical tests.*

Wilcoxon signed rank test: Good: p > 0.05; Poor: ≤ 0.05 [9].

Percentage difference: Good: 0.0 – 10.9%; Acceptable: 11.0 – 20.0%; Poor: > 20.0% [92].

Correlations coefficient (Spearman): Good: ≥ 0.50; Acceptable: 0.20 – 0.49; Poor < 0.20[2]

Cross-classification (Tertiles) (% in same tertile): Good: ≥ 50%; Poor: < 50% [2].

Cross-classification (% in opposite tertile): Good: ≤ 10%, Poor: > 10% [2].

Weighted Kappa statistics: Good: ≥ 0.61; Acceptable: 0.20 – 0.59; Poor: < 0.20 [2].

Bland-Altman - Correlation coefficient (Spearman): Good: P > 0.05; Poor: P ≤ 0.05 [2].

#### Total energy intake

Two interpretations showed good validity (Wilcoxon signed rank test and % difference), two showed acceptable validity (Spearman correlation and weighted Kappa coefficient and three poor validity (cross-classification: % in same & opposite tertiles and Bland Altman analyses).

#### Total protein intake

One interpretation showed good validity (Wilcoxon signed rank test), two acceptable validity (Spearman correlation and % difference) and four poor validity (weighted Kappa coefficient, cross-classification: % in same & opposite tertiles and Bland Altman analyses).

#### Total fat intake

Three interpretations showed good validity (Wilcoxon signed rank test, % difference and Bland Altman) and four showed poor validity (Spearman correlation, cross-classification: % in same & opposite tertiles and weighted Kappa coefficient).

#### Total carbohydrate intake

Three interpretations showed good validity (Wilcoxon signed rank test, % difference and cross-classification: % in same tertile), two showed acceptable validity (Spearman correlation and weighted Kappa coefficient) and two showed poor validity (cross-classification: % in opposite tertile and Bland Altman analyses).

#### Folate intake

Two interpretations showed good validity (cross-classification: % in same & opposite tertiles), two showed acceptable validity (Spearman correlation and weighted Kappa coefficient) and three showed poor validity (Wilcoxon signed rank test, % difference and Bland Altman analyses).

#### Vitamin A intake

All interpretations showed poor validity with the exception of the Bland Altman analyses, which indicated that bias was not present.

#### Iron intake

Two interpretations showed good validity (Wilcoxon signed rank test and cross-classification: % in same tertile), two showed acceptable validity (Spearman correlation and cross-classification: % in opposite tertile) and three showed poor validity (% difference, weighted Kappa coefficint and Bland Altman analyses).

The width of the LOA for total energy, macro and micronutrient intakes can most probably be interpreted as being wide when considered within the context of their respective DRIs. The percentage data points within the LOA is above 95% for all nutrients, with the exception of vitamin A (89.4%) and total energy (93.6%) (data presented in the footnote to Table 4).

## Discussion

Our review demonstrated that six statistical tests and seven accompanying interpretation criteria, as well as 21 combinations of these tests, were used in validation of QFFQs against reference methods. It was evident that each test provided insights into a particular facet of validity, either at group or individual level. Application of all six tests would thus provide comprehensive insight into the validity of a particular dietary assessment method. This will allow for the identification of strengths and limitations of the method in terms of the different facets of validity.

The use of correlation coefficients to determine validity of a dietary assessment method as sole statistical test remains very common (7 of the 60 studies, all published in the past five years). The validation outcomes of these studies would thus only reflect strength and direction of association at individual level. Bland and Altman [72] denote the use of the correlation coefficient as sole test as “a totally inappropriate method.”

Combinations of two tests (21 of the 60 studies) or three tests (20 of the 60 studies) were most commonly used. Combinations typically included a correlation coefficient (association at individual level) and then Bland Altman analyses (agreement and presence and direction of bias at group level) and/or cross-classification (agreement at individual level) and/or the paired *t*-test/Wilcoxon single rank test (agreement at group level). Percent difference, which reflects agreement at group level (size and direction of error), was not commonly used. It is clear that conclusions regarding the validity of a particular dietary assessment method will be limited in terms of those facets of validity that were not assessed. Bearing in mind the limited number of tests used in the majority of the reviewed studies, it is a concern that all studies concluded that the test dietary assessment method was valid for use in the respective populations.

In our view, the finding that none of the reviewed studies that included Bland Altman analyses considered the clinical importance of the width of LOA reflects a general lack of information or guidance or agreement in this regard in the field of nutrition. We propose that the dietary reference intakes (DRI) [85-88] for energy or a particular nutrient should be considered to gain insight into the clinical importance of differences found between dietary methods. However, development of set criteria as to what percentage of the DRI reflects clinically unacceptable LOA is complicated, as the cut-offs may vary from one nutrient to the next, bearing in mind the effects of consumption of inadequate or excessive amounts of the particular nutrient in specific target groups. It may be prudent to follow the recommendation by Hanneman and Faan (2008), [84] namely, to specify clinical differences for specific measures priori for interpretation of clinical importance of bias and LOA bearing in mind the research question and target population. Failure to consider this facet of validity may result in clinically inappropriate conclusions regarding the validity of a dietary assessment method.

Our illustration of the application and interpretation of all six most used statistical tests using a test data set shows that integrative interpretation of the outcomes of multiple statistical tests may be challenging. For example the results show that for *total energy intake* two of the three group level interpretations indicated good validity, while the third interpretation reflected poor validity (presence of bias). Only two of the four individual level interpretations indicated acceptable validity, while two (cross-classification) reflected poor validity. An integrative interpretation of these outcomes for total energy intake could be that the validity of the dietary assessment method is good for total energy intake at group level, bearing in mind that bias may be present. However, support for validity at individual level is not strong. Ranking of individuals e.g. above or below the estimated energy requirement, may thus need to be interpreted with caution. An alternate interpretation could be that further assessments e.g. calculation of energy expenditure and identification of over and under reporters using the Goldberg cut-off points method, need to be conducted and interpreted before a conclusion regarding validity can be made. The same trend in outcomes, and thus outcomes of integrative interpretation, is evident for *fat intake* (all three group level interpretations support good validity, while all four individual level interpretations reflect poor validity) and *protein intake* (two group level interpretations support good validity, while three individual level interpretations reflect poor validity).

Interpretation outcomes for carbohydrate intake show that both group and individual level validity are supported (two group and three individual level interpretations reflect acceptable to good validity). The fact that carbohydrate containing maize based foods are staples of the subjects included in test data sample [12] may have enhanced recall and thus validity outcomes.

Outcomes for *folate and iron* intake show that individual level validity may be more strongly supported than group level validity (folate: all four interpretations at individual level support validity, but not one of the group level interpretations; iron: three individual level and one group level interpretation support validity). These outcomes provide support for validity of ranking of individuals, but not necessarily for comparisons between groups. Confirmation of this conclusion using appropriate biomarkers for folate and iron intake may be necessary.

It is clear that validity of vitamin A intake estimates is not supported, with all interpretations except Bland Altman analyses (no bias present) reflecting poor validity. This outcome may be linked to the likelihood that good sources of vitamin A are not consumed on a daily basis by subjects included in the test data set. It could thus be argued that the QFFQ with a recall period of the past month (test method), may provide a better estimate of usual vitamin A intake than the four 24-hour recalls. In this case it would be prudent to confirm the conclusion of the relative validity outcomes using an appropriate biomarker for vitamin A. The method of triads, a triangular comparison between the test method, the reference method, and biomarker that provides a hypothetical estimate of the validity coefficient of the test method, [89] could be applied for these purposes.

## Conclusions

Our review of dietary assessment method validation studies that involved QFFQs showed that a combination of six statistical tests, namely the T-tests/Wilcoxon rank test, percent difference, correlation coefficients, cross-classification (% in same and opposite tertiles), weighted Kappa coefficients and Bland Altman analyses are used in dietary assessment method validation. The number of statistical tests typically used varies between one and three, which may not be sufficient to provide comprehensive insights into the various facets of validity. The results of our application and interpretation of multiple statistical tests in dietary assessment method validation support the notion that there is value of such an approach in gaining comprehensive insights into and interrogating different facets of validity. These insights should be considered in the formulation of conclusions regarding the validity of the method and decision-making regarding the use of the method to answer a particular dietary intake related research question and subsequently in the interpretation and discussion of the results of the actual research.
